# Identification of a Novel Reference Gene for Apple Transcriptional Profiling under Postharvest Conditions

**DOI:** 10.1371/journal.pone.0120599

**Published:** 2015-03-16

**Authors:** Tatiane Timm Storch, Camila Pegoraro, Taciane Finatto, Vera Quecini, Cesar Valmor Rombaldi, César Luis Girardi

**Affiliations:** 1 Empresa Brasileira de Pesquisa Agropecuária Uva e Vinho, Bento Gonçalves, Brazil; 2 Universidade Federal de Pelotas, Pelotas, Brazil; Virginia Tech, UNITED STATES

## Abstract

Reverse Transcription quantitative PCR (RT-qPCR) is one of the most important techniques for gene expression profiling due to its high sensibility and reproducibility. However, the reliability of the results is highly dependent on data normalization, performed by comparisons between the expression profiles of the genes of interest against those of constitutively expressed, reference genes. Although the technique is widely used in fruit postharvest experiments, the transcription stability of reference genes has not been thoroughly investigated under these experimental conditions. Thus, we have determined the transcriptional profile, under these conditions, of three genes commonly used as reference—*ACTIN (MdACT)*, *PROTEIN DISULPHIDE ISOMERASE (MdPDI)* and *UBIQUITIN-CONJUGATING ENZYME E2 (MdUBC)*—along with two novel candidates—*HISTONE 1 (MdH1)* and *NUCLEOSSOME ASSEMBLY 1 PROTEIN (MdNAP1)*. The expression profile of the genes was investigated throughout five experiments, with three of them encompassing the postharvest period and the other two, consisting of developmental and spatial phases. The transcriptional stability was comparatively investigated using four distinct software packages: BestKeeper, NormFinder, geNorm and DataAssist. Gene ranking results for transcriptional stability were similar for the investigated software packages, with the exception of BestKeeper. The classic reference gene *MdUBC* ranked among the most stably transcribed in all investigated experimental conditions. Transcript accumulation profiles for the novel reference candidate gene *MdH1* were stable throughout the tested conditions, especially in experiments encompassing the postharvest period. Thus, our results present a novel reference gene for postharvest experiments in apple and reinforce the importance of checking the transcription profile of reference genes under the experimental conditions of interest.

## Introduction

Currently, reverse transcription quantitative PCR (RT-qPCR) is considered one of the most sensitive, precise and reproducible techniques to detect specific mRNAs, being employed in a wide range of applications [1]. Frequently, the technique requires the use of two or more constitutively expressed reference genes that are unresponsive to the sampled tissue, exogenous stimuli and experimental conditions [2,3]. However, the transcription of several commonly used reference genes has been demonstrated to be unstable under certain experimental conditions [1,4–7]. Thus, the identification of novel, stably expressed genes is necessary for each given experimental condition [8].

Several statistic analysis tools are available to access transcription stability in qPCR experiments, including geNorm [3], DataAssist (Life Technologies, USA), NormFinder [9] and BestKeeper [10], which employ distinct or similar algorithms to identify the most stably expressed genes under certain conditions.

Transcriptional profiling during postharvest conservation is instrumental to the identification of novel regulatory genes associated to physiological and metabolic conditions affecting viability of a wide range of fruits, including apple (*Malus x domestica* Borkh.), where several modifications responsible for quality loss occur during the period [11,12]. Transcriptional profiling by RT-qPCR in postharvest conditions is dependent on the use of reference genes that are stably transcribed under the distinct experimental conditions for ex planta development and storage. The induction of ethylene production during the period leads to several responses, such as; cell wall degradation and the autocatalytic production of the hormone [13,14]. Moreover, changes in the respiratory rates are also observed [12], culminating, in the latter months of storage in fruit senescence, which is accompanied by alterations in a wide range of cellular functions. Thus, the search for adequate reference genes for postharvest experiments is complex and distinct from other biological situations.

In apple, commonly used reference RT-qPCR genes include *TUBULIN* (*TUB*), *ACTIN* (*ACT*), *UBIQUITIN* (*UBI*), *GLYCERALDEHYDE 3-PHOSPHATE DEHYDROGENASE* (*GAPDH*), *PROTEIN DISULPHIDE ISOMERASE* (PDI), and the coding sequence for the 18S subunit of ribosomal RNA [15–23]. However, commonly used reference genes have been demonstrated to be differentially regulated under given experimental conditions in plants [4–6],and there is scarce information on the transcriptional behavior of classical reference genes in apple. Thus, novel candidate reference genes are highly sought after for fruit development, ripening and storage transcriptional analyses. The coding sequences for proteins involved in nuclear DNA organization and cell cycle control are promising candidates as reference genes, due to the constant maintenance of these vital and essential functions throughout development. Among these proteins, the histones are responsible for DNA condensation, organization and regulation in eukaryotic cells, being responsible to maintain chromatin structure and regulate DNA replication and repair, cell proliferation and gene expression by dynamically modulating the interaction between the nucleic acid and transcription factors [24,25]. The formation of the basic structure of DNA packing, the nucleosome, is mediated by histones H2A, H2B, H3 and H4 [26]. Higher order chromatin structures, also known as stabilized nucleosomes, are produced via linker histone H1 [27]. Molecular chaperones, such as NUCLEOSOME ASSEMBLY PROTEIN 1 (NAP1) [28], act to prevent improper associations between histones and other proteins, or between histones and DNA. The chaperone NAP1 is an integral component of chromatin establishment, maintenance and dynamics in eukaryotes, helping nucleosome assembly and promoting chromatin fluidity, which in turn, control gene expression. Members of the NAP family have been demonstrated to interact with a wide range of cellular factors and are likely to perform additional functions, besides histone assembly [29]. Thus, considering the vital role of the proteins encoded by *H1* and *NAP1*, they are hypothesized to be constitutively transcribed in a wide range of conditions, which prompted us to investigate their transcriptional profile in postharvest experiments with apple to evaluate their potential as RT-qPCR reference genes.

In the current study, we have investigated the transcriptional stability of reference genes frequently used in transcription studies in apple (*ACT*, *UBC* and *PDI*) employing distinct plant organs, fruit developmental stages and ripe fruits kept at room temperature and under long term cold storage, combined with treatments with exogenous ethylene, its inhibitor, 1-methylcyclopropene (1-MCP), and distinct controlled atmosphere conditions. Moreover, two novel candidate reference genes, *HISTONE* 1 (*MdH1*) and *NUCLEOSSOME ASSEMBLY PROTEIN* 1 (*MdNAP1*), were proposed and their transcription stability investigated. Comparative analyses of the results from four software packages for transcription stability determination were performed for all tested experimental conditions. Our results demonstrate that *MdH1* is stably transcribed in a wide range of postharvest conditions and is considered a suitable reference gene for RT-qPCR studies employing ripe apples.

## Material and Methods

Biological samples were collected from a commercial orchard and authorized by the property owner Mr. Nelson Balardin.

### Plant material

Apple biological samples were harvested from a commercial orchard of ‘Gala’ cultivar, clone Baigent, grafted on M.9 rootstock, located in Caxias do Sul, RS, Brazil. Samples were collected as described for each experimental condition, immediately frozen in N_2_ and conserved at −80°C until further processing. Ten tissue samples were combined and evenly split into 12 pools for nucleic acid extraction as described below. Three technical replicates were run for each experiment.

### Experimental conditions

Transcription stability for the candidate genes was investigated in five independent experiments (I to V), consisting of developmental and postharvest conditions. In Experiment I (Plant organs)—fully expanded leaves, flowers at anthesis and green fruits, 60 days after anthesis (DAA), were sampled. For Experiment II (Fruit developmental stages)—samples were harvested from 0 up to 105 DAA, when firmness corresponded to 85 Newton, at 15 day intervals. In Experiment III, transcription profiling was obtained for fruit ripening at room temperature, using apples submitted to 1-methylcyclopropene (1-MCP) treatment and control, untreated fruits, kept at room temperature (RT, 25°C) for 12 days, sampled at two day intervals. For Experiment IV—Ethylene treatment on cold stored apples—the fruits were harvested at physiological maturity, separated into three equivalent samples; one treated with ethylene, one, with 1-MCP and the other, maintained as untreated control. Treated and untreated fruits were subsequently stored under cold storage (CS, temperature of 0 ± 0.5°C and relative humidity of 90 ± 5%) for 180 days, sampled at two month intervals after a period of seven days at RT. Experiment V—Cold storage conditions—employed 1-MCP treated and control fruits submitted to CS combined with distinct controlled atmosphere (CA) conditions (0.5%O_2_, 1.0% O_2_ and 1.5% O_2_, supplemented with 2% CO_2_ in all conditions) for nine months. Apples were sampled immediately after removal from CS/CA and after seven days of RT incubation.

### Ethylene and 1-MCP treatments

Exogenous ethylene application in Experiment IV was carried out by supplying fruits contained in hermetically closed flasks with 10 ppm (10μL. L^−1^) of ethylene for four hours. For 1-MCP treatments in Experiments III, IV and V, 1 ppm (1μL. L−1) of the commercial product SmartFresh (Agro Fresh, Rohm and Haas, PA, USA), containing 0.14% of the active principle, was applied to water and the resultant gas was applied to the fruits for 24 hours in hermetically closed chambers.

### Total RNA isolation and first strand cDNA synthesis

Total RNA was isolated as described by Zeng and Yang [30], with an additional precipitation step with sodium acetate 3 M pH 5.5, followed by incubation at −80°C for 25 min and centrifugation at 20,000 x g for 20 min at 4°C, before the overnight precipitation with 10 M LiCl. Quality and quantity of the RNA were analyzed by absorbance ratio at A_260_/A_280_ and A_260_/A_230_ using an Epoch Micro-volume (BioTek, VT, USA) spectrophotometer. The integrity of the total RNAs was investigated by 1% agarose gel electrophoresis, stained with Gel Red (Biotium, CA, USA). Genomic DNA was eliminated by DNAse I (Invitrogen, MA, USA) treatment, as recommended by the manufacturer. The efficiency of DNA removal was checked by qPCR using *MdH1* and *MdUBC* primers, as described. Subsequently, cDNA synthesis was carried out from 1 μg of total RNA using Oligo d(T) (Invitrogen) primers and SuperScriptIII/RNAse Out Mix (Invitrogen), according to the manufacturer recommendations.

### Primer design and RT-qPCR conditions

Primers were designed for coding sequences (CDs) of the candidate reference genes of *Malus x domestica* genome, available at the Genome Database for Rosaceae (GDR, - http://www.rosaceae.org/), using the default parameters of the software Primer3Plus [31], with amplicon sizes ranging from 80 to 150 base pairs (Table 1). The primers were validated for each experiment by the analyses of the amplification curve employing a pool of cDNAs from all tested conditions, at five distinct concentrations. Oligonucleotide specificity and absence of primer dimers were checked by analyses of the post transcriptional dissociation curve. All primers used in the current study exhibited amplification efficiency close to 2.0 and a single dissociation temperature peak (S1 Fig.).

**Table 1:** Genome and amplification information on the candidate reference genes.

		Primer Sequence (5′to 3′)		Amplicon	Temperature of melting (°C)	
Gene description/ acronym	Accession code^a^	Forward	Reverse	size (bp)	Forward	Reverse
*PROTEIN DISULFIDE ISOMERASE* (*PDI*)	MDP0000233444	TGCTGTACACAGCCAACGAT	CATCTTTAGCGGCGTTATCC	120	60	60
*HISTONE 1* (*H1*)	MDP0000223691	CATATTTGGCAGCAGAGCAA	CTCGTTAGCCAACTGCATCA	89	58	60
*NUCLEOSSOME ASSEMBLY 1 PROTEIN* (*NAP1*)	MDP0000272485	CAAACTTGCCCCTCCATTTA	CCAGCCTTCGTGATGAATTT	117	58	58
*UBIQUITIN-CONJUGATING ENZYME E2* (*UBC*)	MDP0000205182	TTGCTGGTGATCTCTGCATC	AGACCCACCTACTCCCGTCT	117	60	60
*ACTIN* (*ACT*)	MDP0000170174	GGCTCTATTCCAACCATCCA	TAGAAGCAGTGCCACCACAC	140	60	62

^a^ Accession codes correspond to unigene numbers from *M*. x *domestica* genome.

Quantitative PCR (qPCR) was carried out using the equipment StepOne Real Time PCR Systems (Life Technologies, MA, USA) and the SYBR Green PCR Master Mix (Life Technologies). The reactions were performed in a total volume of 15 μL, consisting of 7.5 μL of SYBR Green PCR Master Mix, 1 μL of cDNA and 400nM each, forward and reverse, primer, and started with a denaturation step at 95°C for 10 minutes, followed by 40 cycles consisting of 15 seconds at 95°C and 1 minute at 60°C, finalized by the dissociation curve with denaturation at 95°C for 15 seconds, cooling at 60°C for 1 minute and gradual heating, at 0.3°C steps, up to 95°C. A negative, no template control (NTC), was used to check the absence of DNA contamination.

We investigated the transcriptional stability of five candidate reference genes, consisting of three commonly used normalizers; *ACTIN* (*MdACT*), *UBIQUITIN-CONJUGATING ENZYME E2* (*MdUBC*), and *PROTEIN DISSULFIDE ISOMERASE* (*MdPDI*), and two novel proposed candidates; *HISTONE 1* (*MdH1*) and *NUCLEOSSOME ASSEMBLY 1 PROTEIN* (*MdNAP1*).

### Statistical analyses of transcriptional stability

Quantification cycle (Cq) data from RT-qPCR were recovered using the software StepOne v 2.2.2 (Applied Biosystems, MA, USA) and submitted to analyses using four software packages, namely DataAssist v3.01 (Life Technologies), geNorm [3], NormFinder [9] and BestKeeper [10].

The BestKeeper algorithm employs gross Cq values to calculate the standard deviation (SD) and coefficient of variation (CV) for each candidate under the investigated experimental conditions. Subsequently, it calculates a ‘BestKeeper Index’ for each sample, consisting of the geometric mean of the Cq values for each candidate reference gene. Finally, the program calculates a Pearson coefficient of correlation (*r*) between the candidate reference gene and the BestKeeper Index, where high *r* values correspond to genes exhibiting stable transcriptional profile under the investigated experimental conditions [10].

The geNorm package ranks the transcriptional stability of the candidate genes by the M value, which is calculated by the pairwise variation in the transcript levels of a given gene in comparison to the levels of the remaining reference genes in test. Gross Cq values were converted to linear data for M calculations [3]. Genes exhibiting high M values are considered transcriptionally unstable under the tested conditions. The M value is then used to stepwise exclude the genes exhibiting the most unstable transcriptional profiles and recalculate the M values for the remaining genes. Genes with M values inferior to 1.5 are considered stably transcribed in the investigated conditions.

The package DataAssist employs an algorithm similar to that of geNorm to evaluate the transcription stability of the candidate genes, by generating a score for each candidate reference gene that is inversely proportional to its transcriptional stability.

In contrast, NormFinder uses an ANOVA based model to investigate the transcriptional behavior of a candidate reference throughout the tested experimental conditions. Inter and intragroup variation is used to rank the transcriptional stability of the candidate reference genes. Thus, lower variation values correspond to the most stably transcribed candidates [9].

## Results

In the current work, we have investigated the transcriptional behavior of five candidate reference genes for RT-qPCR, consisting of three commonly used genes (*MdACT*, *MdUBC* and *MdPDI*) and two novel proposed (*MdH1* and *MdNAP1*) normalizers (Table 1). The transcriptional stability of the candidates in five independent experiments was evaluated using four software packages; geNorm, Normfinder, BestKeeper and DataAssist.

### Amplification efficiency of the selected primers

Specific amplification was confirmed by the presence of a single temperature peak in the dissociation curves of all tested primers (S1 Fig.). The efficiency of amplification for the primers in RT-qPCR reactions ranged from 1.90 to 2.34 for all investigated experimental conditions, with most values close to 2.0 (Table 2).

**Table 2:** Efficiency of primer pairs used for RT-qPCR amplification in each experiment.

	RT-qPCR eficiency (%)				
Gene	Plant organs	Fruit developmental stages	Fruit ripening at room temperature	Ethylene treatment on cold stored apples	Cold storage conditions
*ACT*	1.98 (98.17%)	2.08 (110.74%)	2.10 (110.79%)	2.01 (100.73%)	2.03 (103.92%)
*H1*	1.96 (96.71%)	2.09 (109.77%)	2.04 (104.53%)	2.05 (105.93%)	2.00 (100.39%)
*NAP1*	1.95 (95.19%)	1.92 (92.51%)	1.94 (94.88%)	2.10 (110.65%)	2.22 (122.83%)
*PDI*	1.95 (94.64%)	2.09 (109.46%)	2.07 (107.91%)	2.08 (108.71%)	2.17 (116.65%)
*UBC*	1.91 (91.47%)	2.01 (101.53%)	2.07 (107.31%)	2.00 (101.10%)	1.98 (98.27%)

The genes were investigated under five independent conditions, using a pool of the cDNAs for all experimental conditions, at five distinct concentrations.

### Expression levels of candidate reference genes

**Fig 1:**
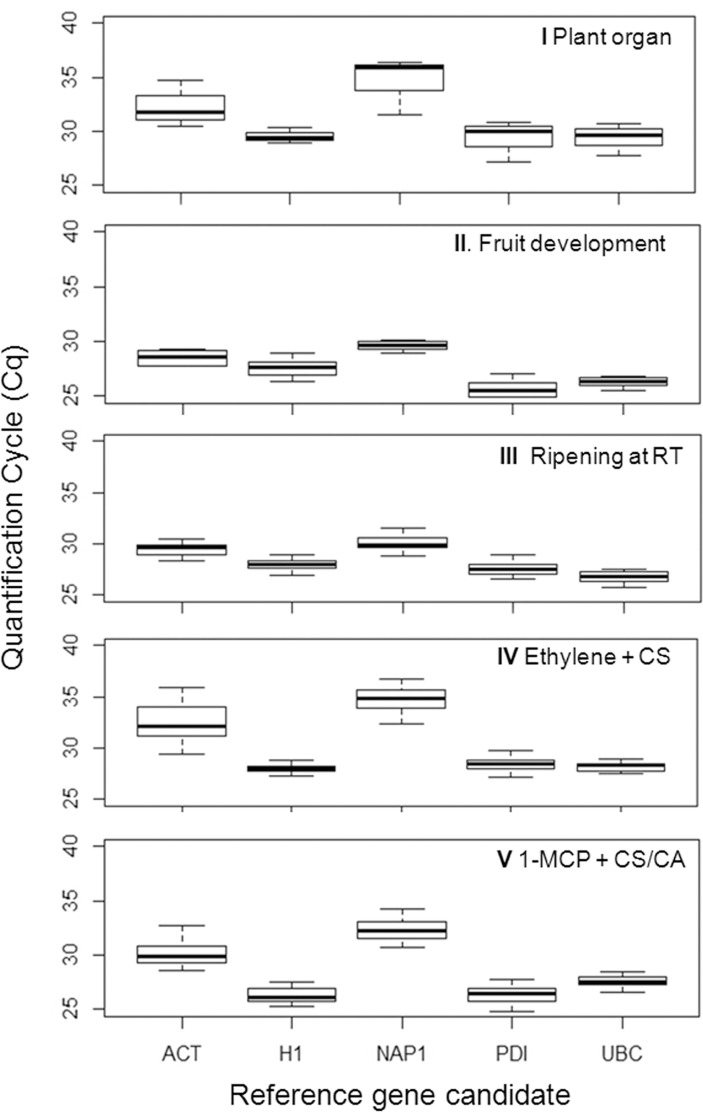
Gene expression levels of the candidate reference genes in apple. The genes, *MdACT* (*ACTIN*), *MdH1* (*HISTONE1*), *MdNAP1* (*NUCLEOSSOME ASSEMBLY PROTEIN* 1), *MdPDI* (*PROTEIN DISULPHIDE ISOMERASE)* and *MdUBC* (*UBIQUITIN-CONJUGATING ENZYME E2*) were evaluated in five independent experiments consisting of plant organs (I), fruit developmental stages (II), fruit ripening at room temperature (III), ethylene treatment on cold stored apples (IV) and long term cold storage combined with distinct controlled atmosphere conditions (V). Horizontal bar represents Cq median and whiskers represent highest and lowest values.

Transcriptional stability is dependent on the Cq values of the candidate reference genes throughout the tested experimental conditions. For novel candidate *MdNAP1*, Cq values were highly variable, ranging from 28 to 37 in the investigated experimental conditions (Fig. 1). The most variable levels of transcription for *MdNAP1* were observed in response to ethylene treatment applied to cold stored apples (Experiment IV) (Fig. 1). Transcript accumulation for *MdACT*, a commonly employed normalizer gene in RT-qPCR experiments, was also variable for all investigated experimental conditions (Fig. 1). The Cq values for these candidates were also higher than those observed for the remaining potential references, demonstrating the low transcription rates of the genes in the evaluated samples (Fig. 1). In a similar manner, the highest Cq values of 37 and 36 for *MdNAP1* and *MdACT*, respectively, were observed in response to ethylene treatment of cold stored apples (Experiment IV) (Fig. 1). In contrast, the smallest variation of Cq values in the tested experimental conditions were observed for *MdH1* and *MdUBC* (Fig. 1). The highest transcription activation was observed for *MdPDI*, in response to fruit ripening stage (Experiment II), with Cq ranging from 24 to 27 (Fig. 1).

### Transcription stability analyses

#### BestKeeper

The results of BestKeeper analyses indicate distinct sets of genes as the most suitable references for each experimental condition tested. The transcription of the candidates was highly responsive to the type of plant organ investigated (Experiment I), with the exception of *MdH1* that exhibited SD smaller than 1 and was considered a suitable reference gene for transcriptional profiling in plant organs (Table 3, S1 Table). The values of CV were also high for *MdNAP1* (5.90), *MdACT* (5.01), *MdPDI* (4.94) and *MdUBC* (3.63) expression in distinct plant parts. In contrast, for fruit developmental stage (Experiment II), SD values for all investigated candidate genes were inferior to 1.0, with the smaller variations being found in *MdUBC* and *MdNAP1* transcript accumulations (Table 3). A similar trend of low SD values was also observed for apple fruits ripening at room temperature (Experiment III), with *MdH1* and *MdUBC* exhibiting the lowest SDs (Table 3). The transcript accumulation of *MdACT* and *MdNAP1* was responsive to ethylene treatment in cold stored fruits (Experiment IV), which exhibited SDs superior to 1.0 and CVs of 5.22 and 3.89, respectively. As observed for Experiment III, *MdH1* and *MdUBC* were the least responsive genes in Experiment IV (Table 3). Similarly, the transcript accumulation profile for both genes was also the most stable in response to distinct controlled atmosphere conditions in cold stored fruits (Experiment V). In contrast, SD value for *MdACT* expression in Experiment V was higher than 1.0 and CV was of 4.32; therefore, the gene was considered unsuitable as reference for studies of apple fruits under controlled atmosphere combined with cold storage (Table 3).

**Table 3:** Ranking of the five candidate reference genes according to the transcription stability.

Software	DataAssist		NormFinder		geNorm		BestKeeper	
	Score	Ranking	Stability value	Ranking	M value	Ranking	Coeff. of. corr. [r]	Ranking
I. Plant organs	1.16	*PDI*	0.178	*PDI*	1.058	*PDI*	-	-
	1.17	*UBC*	0.178	*UBC*	1.076	*UBC*	-	-
	1.52	*ACT*	0.930	*ACT*	1.586	*ACT*	-	-
	2.00	*NAP1*	0.964	*NAP1*	1.653	*NAP1*	-	-
	2.04	*H1*	1.129	*H1*	1.823	*H1*	-	-
II. Fruit developmental stages	0.45	*ACT*	0.073	*PDI*	0.459	*ACT*	0.974	*PDI*
	0.46	*PDI*	0.087	*ACT*	0.463	*PDI*	0.957	*ACT*
	0.48	*UBC*	0.155	*UBC*	0.489	*UBC*	0.949	*UBC*
	0.66	*NAP1*	0.417	*NAP1*	0.666	*NAP1*	0.817	*NAP1*
	0.73	*H1*	0.467	*H1*	0.732	*H1*	0.795	*H1*
III. Fruit ripening at room temperature	0.65	*H1*	0.242	*H1*	0.688	*H1*	0.916	*NAP1*
	0.65	*UBC*	0.309	*ACT*	0.726	*ACT*	0.748	*H1*
	0.69	*ACT*	0.343	*UBC*	0.732	*UBC*	0.741	*UBC*
	0.83	*NAP1*	0.501	*NAP1*	0.885	*NAP1*	0.734	*ACT*
	0.85	*PDI*	0.501	*PDI*	0.891	*PDI*	0.611	*PDI*
IV. Ethylene treatment on cold stored apples	1.42	*UBC*	0.308	*UBC*	0.980	*UBC*	0.986	*UBC*
	1.50	*H1*	0.461	*H1*	1.085	*H1*	0.913	*PDI*
	1.57	*PDI*	0.618	*PDI*	1.148	*PDI*	0.853	*H1*
	2.03	*ACT*	0.756	*NAP1*	1.434	*NAP1*	-	-
	2.57	*NAP1*	1.033	*ACT*	1.660	*ACT*	-	-
V. Cold storage conditions	1.01	*UBC*	0.065	*UBC*	1.015	*UBC*	0.883	*H1*
	1.10	*PDI*	0.343	*PDI*	1.102	*PDI*	0.856	*PDI*
	1.11	*H1*	0.490	*H1*	1.110	*H1*	0.817	*UBC*
	1.27	*NAP1*	0.708	*NAP1*	1.274	*NAP1*	0.754	*NAP1*
	1.69	*ACT*	1.097	*ACT*	1.690	*ACT*	-	-

Ranking parameter is presented for each software. Absent data (-) corresponds to the lack of suitable ranking parameters, indicating unstable transcription under the tested conditions.

Genes exhibiting variable transcription levels (high SDs) also displayed strong correlations (*r*), indicating co-regulation. As recommended, the genes with SD greater than 1.0 were eliminated from further analyses in Experiment I. Thus, *MdH1* was the sole gene exhibiting acceptable SD value in response to plant organ types (Experiment I). In contrast, for transcription stability in several fruit ripening stages (Experiment II), the results of the full analysis (candidate gene ranking by coefficient of correlation) were similar to those observed for the initial classification based on SD (Table 3, S1 Table). The correlation between the expression and BestKeeper indices was strong and significant for *MdPDI*, *MdACT* and *MdUBC* in response to the fruit developmental stage, whereas for *MdH1*, *r* was not significant (Table 3). Significant coefficients of correlation were also observed for *MdUBC*, *MdPDI* and *MdH1* in response to ethylene treatment of cold stored fruits (Experiment IV). Under distinct CA conditions for fruits kept in CS (Experiment V), the most stably transcribed genes were *MdH1*, *MdPDI* and *MdUBC*, and the least stable transcription levels were observed for *MdNAP1* (Table 3, S1 Table).

#### geNorm

**Fig 2:**
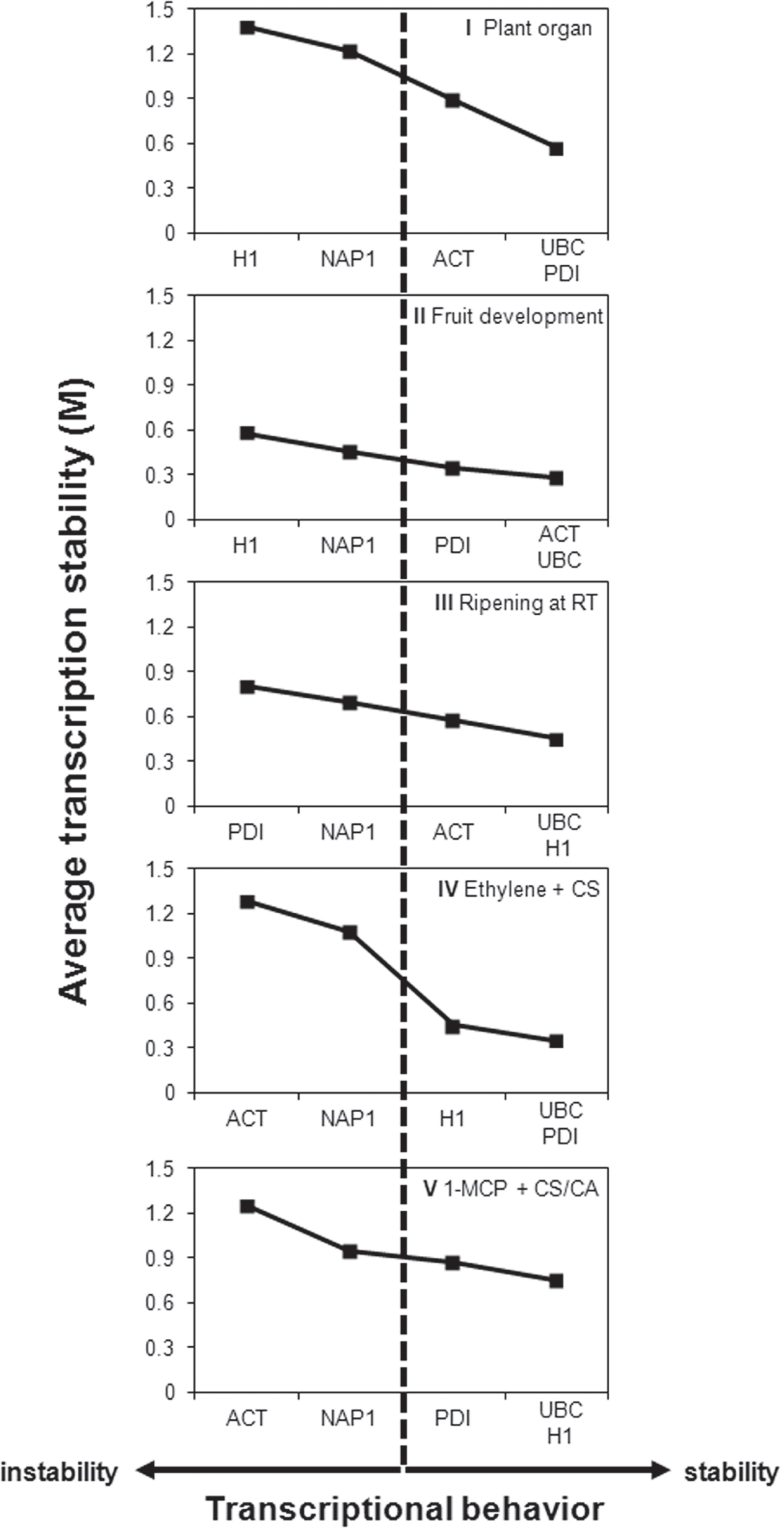
Transcriptional stability of the candidate reference genes investigated by geNorm. The candidate reference genes, *MdACT* (*ACTIN*), *MdH1* (*HISTONE1*), *MdNAP1* (*NUCLEOSSOME ASSEMBLY PROTEIN* 1), *MdPDI* (*PROTEIN DISULPHIDE ISOMERASE)* and *MdUBC* (*UBIQUITIN-CONJUGATING ENZYME E2*) were submitted to five experimental conditions, consisting of plant organs (I), fruit developmental stages (II), fruit ripening at room temperature (III), ethylene treatment on cold stored apples (IV) and long term cold storage combined with distinct controlled atmosphere conditions (V). Low M values indicate genes with more stable transcript accumulation under the given conditions.

The results of M value analyses using geNorm software demonstrated that not all candidates are suitable reference genes in all tested experimental conditions (Table 3). Genes considered transcriptionally unstable (M > 1.5) were *MdACT* in Experiments I, IV and V, and *MdNAP1* and *MdH1* in Experiment I (Table 3). The recommended stepwise exclusion of unsuitable reference genes reduced M values for all investigated genes in all tested experimental conditions (Fig. 2).

The initial classification, based on M values, and the final classification, after the stepwise exclusion of the genes exhibiting higher M values, resulted in slightly different order of the recommended normalizers for Experiments II, III, IV and V (Table 3, Fig. 2).

#### DataAssist

The analysis algorithm for DataAssist is similar to that of geNorm, with the recommendation of most stably transcribed genes based on a calculated score. For Experiments I and II (plant organs and fruit developmental stages), the genes exhibiting the most variable patterns of transcript accumulation were *MdH1* and *MdNAP1*, whereas *MdPDI*, *MdACT* and *MdUBC* were considered to have the most stable transcript accumulation levels (Table 3). The least responsive genes to 1-MCP treatment of room temperature stored apples (Experiment III) were *MdH1*, *MdUBC* and *MdACT*. Similarly, *MdUBC* and *MdH1*, along with *MdPDI*, were stably transcribed in fruits treated with ethylene and cold stored (Experiment IV) (Table 3). The same three candidate reference genes were also recommended as the most adequate normalizers for fruits kept under CS in different CA conditions (Experiment V), however, in a different order (*MdUBC* < *MdPDI* < *MdH1*) (Table 3).

#### NormFinder

The ANOVA based algorithm of NormFinder generated a similar rank of recommended reference genes for transcriptional analyses using distinct plant organs (Experiment I) (Table 3). The classification of the most stably transcribed genes in response to the fruit developmental stages (Experiment II) was similar for all employed software packages, except that the recommended order by geNorm and DataAssist was slightly different than the order recommended by NormFinder and BestKeeper (Table 3). The genes exhibiting the most unresponsive transcriptional profiles to 1-MCP treatment and storage at RT (Experiment III) according to NormFinder analyses were *MdH1*, *MdACT* and *MdUBC*, in agreement with the results from the other tested packages, which also detected the transcription instability of *MdNAP1* and *MdPDI* under these experimental conditions (Table 3). The least stably transcribed genes in response to ethylene treatment under cold storage in NormFinder analyses were *MdNAP1* and *MdACT* and similar results were generated by the other packages (Table 3). Under distinct CA conditions in cold storage, the most stable levels of transcript accumulation were observed for *MdUBC*, *MdPDI* and *MdH1*, in agreement with the results with DataAssist (Table 3).

## Discussion

The transcriptional stability of five candidate genes, consisting of three commonly used and two novel proposed references, for transcriptional profiling was investigated in apple by the comparative analyses of the results generated using four software packages with data from five experiments consisting of developmental and postharvest conditions. Recently, the transcriptional stability of a wide range of reference genes has been investigated in plants [4–7,32–35]. Taken together, the results suggest that transcriptional stability is dependent on the biological material and the experimental conditions to which it has been submitted, and that the use of two or more reference genes is recommended for adequate normalization. To our knowledge, the present work is the first to investigate the transcriptional behavior of reference genes in apple postharvest studies.

Distinct calculation tools are available to investigate the transcriptional stability of candidate reference genes [3,9,10]. We have employed four software packages, namely; geNorm, NormFinder, BestKeeper and DataAssist, to study the transcript accumulation profile of the candidates in five independent experiments. Although some packages recommend a certain number of reference genes to be used for a given experimental situation [3], the use of three reliable references is suitable to normalize a wide range of qPCR applications [10,36]. The comparative analysis of the transcription stability results by the four software packages has demonstrated minor differences in candidate gene ranking (Table 3), which is a consequence of the algorithms used by each program. Although the ranking order was not always identical, the three genes considered the most stably transcribed under a given experimental condition were similar. The noteworthy exception was the rank generated by BestKeeper for fruits treated with 1-MCP and stored at RT (Experiment III), which classified *MdNAP1* as the gene exhibiting the most stable transcription pattern, whereas the results obtained from the remaining packages ranked the gene amongst the least transcriptionally stable under those experimental conditions. Moreover, the initial ranking by BestKeeper, based on SD, suggests *MdH1* as a suitable reference gene for transcriptional profiling using distinct plant organs (Experiment I) (S1 Table), in contrast with the ranks generated by the other packages that considered *MdH1* differentially regulated in response to the experimental conditions. Thus, in general, the most discrepant results in gene stability ranking were obtained with BestKeeper. The most critical difference between BestKeeper algorithm and those from the other packages is that it employs the correlation analyses between the candidate genes Cq and an index derived from the candidate geometric mean. In contrast, the algorithms used by geNorm, DataAssist and NormFinder employ variation measures to calculate the stability of gene transcription [3,9,10]. Under our experimental conditions, the software BestKeeper was not suitable to precisely determine the most stably transcribed genes, since no results were generated for Experiment I (plant organs) and the results obtained for Experiment III (fruit ripening at room temperature) were divergent from the results given by the other packages. A comparative analyses of transcriptional stability in distinct tissues of pear also reported similar results obtained by geNorm and NormFinder analyses [33].

Fruit storage and postharvest periods are characterized by metabolic alterations distinct from those occurring during other developmental stages. Moreover, several particular metabolic aspects are typical of fruits and absent from vegetative tissue and flower development. Thus, the transcriptional profile of the candidate reference genes was investigated in distinct plant organs and throughout fruit development. In agreement with these observations, the transcript accumulation of the gene *MdH1* was stable in all experimental conditions involving ripe, harvested fruits (Experiments III, IV and V), whereas, its transcription was differentially regulated in the tested plant organs and during fruit development, before physiological maturity (Experiments I and II) (Fig. 1, Table 3). Although histone coding sequences, such as those coding for H2 and H3, have been used as reference genes in qPCR studies with several organisms [33,37–39], the current work is the first one to propose an H1 coding sequence as reference. Histone 1, also called Histone 5, is classified as a linker histone due to its function in the association of nucleosome core particles during chromatosome formation. The linker function is distinct from that of H2A, H2B, H3 and H4 that constitute the core histone octamer around which the DNA is wrapped during chromatin condensation [25]. Recently, the occupancy of histone variants in nucleosomes has been associated to environmental and developmental control in higher plants; including processes associated to temperature and senescence responses [40,41]. The observed transcriptional stability of *MdH1* suggests that it does not participate in these processes in mature apples. Future studies investigating the transcriptional regulation of H1 coding sequences in other fruit species or even apple genotypes may provide further insight into its biological role and usefulness as reference gene in transcription profiling investigation.

The transcriptional stability of *MdACT*, a commonly employed reference gene, was observed in the majority of the investigated experimental conditions, except for experiments IV and V, which use cold storage for postharvest fruit conservation. Similarly, previous studies have demonstrated that *ACT* transcription in tissues submitted to cold is less stable [42,43], suggesting that low temperatures influence its transcription and/or turnover rates. The transcription stability of the gene *MdUBC* was observed for all investigated experimental conditions; thus, it can be considered a suitable reference gene for a wide range of biological samples and experimental conditions. Moreover, results from the analyses with the four software packages tested included *MdUBC* among the three most stably transcribed genes throughout the experimental conditions. The novel proposed candidate *MdNAP1* ranked amongst the least stably transcribed genes in the majority of the investigated experimental conditions. Therefore, it was considered inadequate as a reference gene for the tested conditions. The low transcriptional stability may be associated to the low levels of transcript accumulation detected under our experimental conditions, suggesting that its transcription is significantly affected by ethylene, ethylene-triggered signal transduction and/or low temperatures.

## Conclusions

The gene *MdH1* constitutes a novel, viable, alternative for RT-qPCR data normalization in postharvest studies with apple fruits.

The ranking results of transcription stability analyses employing the software BestKeeper were the most divergent, among those from the investigated packages.

Taking together, the results from the present work reinforce the importance of the determination of transcription stability for the experimental conditions and candidate reference genes of interest.

## Supporting Information

S1 Fig Primer amplification specificity for the candidate reference genes. These results were obtained by RT qPCR dissociation curves, for the primers used to investigate the transcription of the candidate reference genes in five independent experiments (I to V): (I) plant organ; (II) fruit developmental stages; (III) fruit ripening at room temperature; (IV) ethylene treatment on cold stored apples and (V) long term cold storage combined with distinct controlled atmosphere conditions.
(TIF)

S1 Table Descriptive statistic analyses by BestKeeper. The transcriptional regulation of five candidate reference genes was tested in five independent experimental conditions in apple. Absent data (-) corresponds to unviable calculations due to differentially regulated transcription under the given experimental conditions.
(PDF)
