# Supplementary figures and images for: Identification of a Novel Reference Gene for Apple Transcriptional Profiling under Postharvest Conditions
Source: PLoS One. 2015 Mar 16;10(3):e0120599. doi: 10.1371/journal.pone.0120599 (PMC4361542; doi:10.1371/journal.pone.0120599)

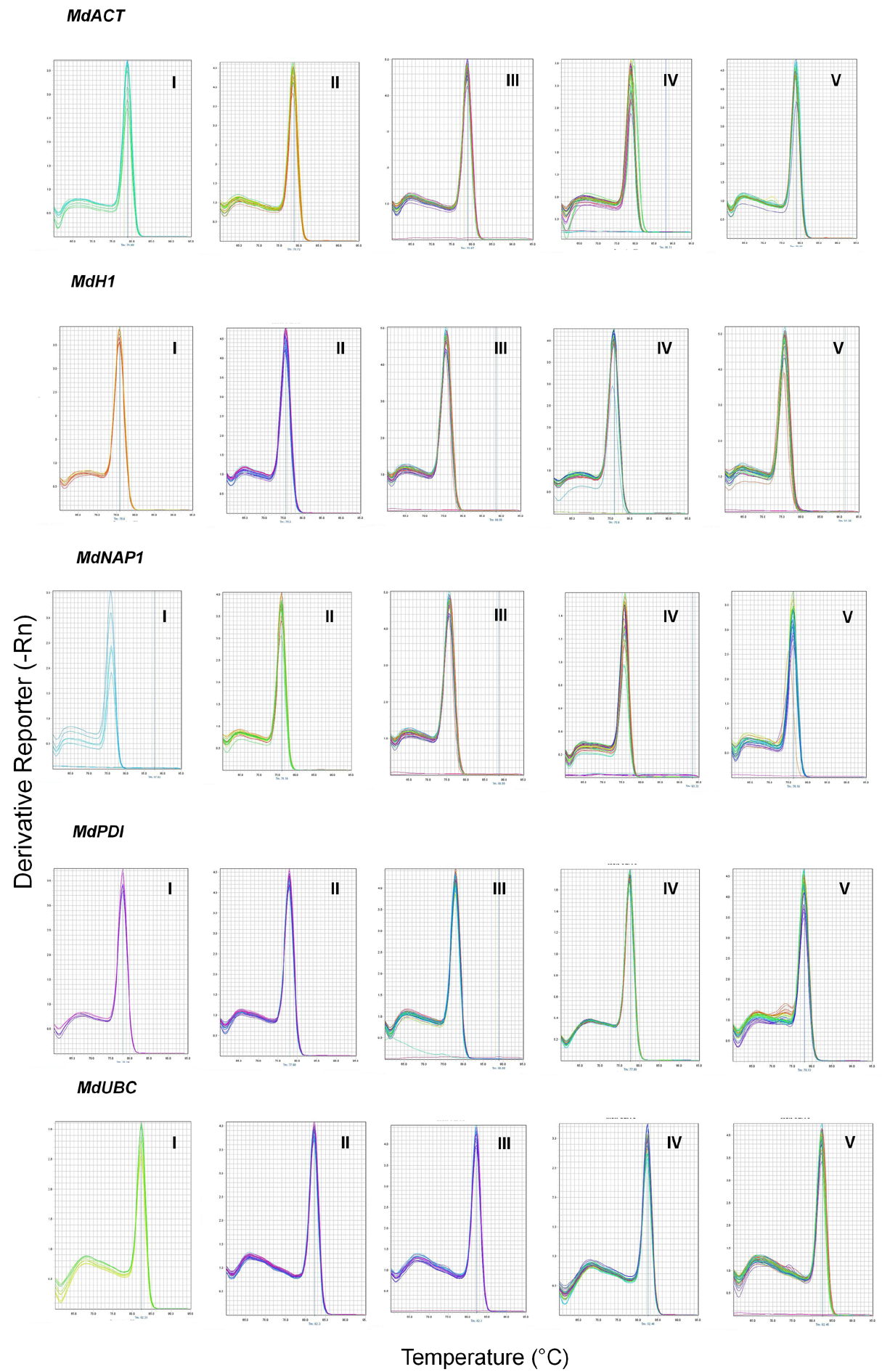

Supplement: S1 Fig — These results were obtained by RT qPCR dissociation curves, for the primers used to investigate the transcription of the candidate reference genes in five independent experiments (I to V): (I) plant organ; (II) fruit developmental stages; (III) fruit ripening at room temperature; (IV) ethylene treatment on cold stored apples and (V) long term cold storage combined with distinct controlled atmosphere conditions. (TIF) [file pone.0120599.s001.tif]
