# Supplementary material for: Identification of a Novel Reference Gene for Apple Transcriptional Profiling under Postharvest Conditions
Source: PLoS One. 2015 Mar 16;10(3):e0120599. doi: 10.1371/journal.pone.0120599 (PMC4361542; doi:10.1371/journal.pone.0120599)
Supplement: S1 Table — The transcriptional regulation of five candidate reference genes was tested in five independent experimental conditions in apple. Absent data (-) corresponds to unviable calculations due to differentially regulated transcription under the given experimental conditions. (PDF) [file pone.0120599.s002.pdf]

Supplementary Table 1. Descriptive statistic analyses of the transcriptional regulation of five candidate reference genes tested in five independent experimental conditions in apple, using the software BestKeeper. Absent data (-) corresponds to unviable calculations due to differentially regulated transcription under the given experimental conditions.

Experiment	Plant organs		Fruit developmental stage		Fruit ripening at room temperature		Ethylene treatment on cold stored apple		Cold storage conditions	
	coeff. of corr. [r]	p-value	coeff. of corr. [r]	p-value	coeff. of corr. [r]	p-value	coeff. of corr. [r]	p-value	coeff. of corr. [r]	p-value
**Candidate reference genes**										
ACT	0.863	0.338	0.957	0.001	0.734	0.003	0.893	0.001	0.722	0.001
H1	0.595	0.594	0.795	0.001	0.748	0.002	0.763	0.010	0.750	0.001
NAP1	0.995	0.191	0.817	0.001	0.916	0.001	0.920	0.001	0.590	0.006
PDI	1.00	0.003	0.974	0.001	0.611	0.020	0.605	0.006	0.900	0.001
UBC	0.987	0.104	0.949	0.001	0.741	0.002	0.861	0.001	0.890	0.001
**Candidate reference genes SD<1**										
ACT	-	-	0.957	0.001	0.734	0.003	-	-	-	-
H1	-	-	0.795	0.001	0.748	0.002	0.853	0.002	0.883	0.001
NAP1	-	-	0.817	0.001	0.916	0.001	-	-	0.754	0.001
PDI	-	-	0.974	0.001	0.611	0.020	0.913	0.001	0.856	0.001
UBC	-	-	0.949	0.001	0.741	0.002	0.986	0.001	0.817	0.001
